# The complete chloroplast genome sequences of *Pogostemon stellatus* and *Pogostemon yatabeanus* (Lamiaceae)

**DOI:** 10.1080/23802359.2016.1192509

**Published:** 2016-08-21

**Authors:** Dong-Keun Yi, Ki-Joong Kim

**Affiliations:** School of Life Sciences, Korea University, Seoul, Korea

**Keywords:** Chloroplast genome, Lamiaceae, *Pogostemon stellatus*, *Pogostemon yatabeanus*

## Abstract

The nucleotide sequences of the two chloroplast (cp) genomes from *Pogostemon stellatus* and *Pogostemon yatabeanus* are the first to be completed in genus *Pogostemon* of family Lamiaceae. The structure of two *Pogostemon* cp genomes shows similar characteristic to the typical cp genome of angiosperms. The lengths of two cp genomes are 151,825bp and 152,707 bp, respectively. Two cp genomes are divided into LSC region (83,012 bp and 83,791 bp) and SSC region (17,524 bp and 17,568 bp) by two IR regions (25,644 bp and 25,674 bp). Both of two cp genomes contain 114 genes (80 protein-coding genes, 30 tRNA genes and 4 rRNA genes), 10 protein-coding genes and 7 tRNA genes duplicated in the IR regions. Similar to the typical cp genome of angiosperms, 18 of the genes in the *Pogostemon* cp genome have one or two introns. The overall A-T contents of two genomes are 61.8% which is also similar to general angiosperms. The A-T content in the non-coding (64.4%) is higher than in the coding (59.9%) regions. Sixty-seven and seventy-three simple sequence repeat (SSR) loci were identified in the *P. stellatus* and *P. yatabeanus* cp genomes, respectively. In phylogenetic analysis, genus *Pogostemon* shows closed relationship with *Scutellaria baicalensis* of Scutellarioideae

## Results and discussion

Genus *Pogostemon* which included about 94 species is a large group of the Lamioideae clade in the family Lamiaceae (Panigrahi 1984; Chuakul 2002). None of the cp genome sequence have been published so far in genus *Pogostemon*, therefore we performed cp genome sequencing of *Pogostemon stellatus* (Lour.) Kuntze and *Pogostemon yatabeanus* (Makino) Press. *Pogostemon stellatus* and *P. yatabeanus* are aquatic perennial herbs which are highly acclaimed for ornamental plant in the aquarium trade. Specially, *P. yatabeanus* is one of the endangered species in Korea. The plant materials of *P. stellatus* and *P. yatabeanus* were collected from a single individual that lives in the natural wetland habitats in Jeju-island, South Korea. Voucher specimens and DNA samples were deposited in the Korea University Herbarium (KUS2014-1557, KUS2014-1822) and Plant DNA bank in Korea (PDBK 2014-1557, PDBK 2014-1822), respectively. Chloroplast genome sequences were analyzed using Illumina MiSeq (San Diego, CA), and assembled by Geneious 8.1.6 (Auckland, New Zealand) (http://www.geneious.com, Kearse et al. 2012). The complete cp genome sequences were submitted into NCBI database under the accession number of KP718620 for *P. stellatus* and KP718618 for *P. yatabeanus*, respectively. The lengths of complete cp genome sequence of *P. stellatus* and *P. yatabeanus* are 151,825bp and 152,707bp, respectively. The cp genome of *P. stellatus* is composed of 83,012bp of LSC region, 17,524bp of SSC region and 25,644bp of two IR regions, whereas the cp genome of *P. yatabeanus* is composed of 83,791bp of LSC region, 17,568bp of SSC region and 25,674bp of two IR regions. Both of two cp genomes consist of 114 individual genes which included 80 protein-coding genes, 30 transfer RNA genes and 4 ribosomal RNA genes. Among them, 10protein-coding genes and 7 tRNA genes are duplicated on the IR regions. Similar to the typical cp genome of angiosperms such as *Panax* and *Sesamum*, 18 of the genes in the each cp genome have one or two introns (Kim & Lee 2004; Yi & Kim 2012). Of these, rps12, clpP and ycf3 have two introns.

The major portion of the *P. stellatus* and *P. yatabeanus* cp genomes consist of gene-coding regions (59.2% and 58.9%) which consist of protein-coding region (52.8% and 52.6%) and RNA regions (6.4% and 6.3%), whereas the intergenic spacers (including 23 introns) comprise 40.8% and 41.1%, respectively. The overall A-T contents of two genomes are 61.8% which is similar to general angiosperms and other cp genomes of Lamiaceae (Shinozaki et al. 1986; Kim & Lee 2004; Cronn et al. 2008; Yi & Kim 2012; Zhu et al. 2014). In both of two genomes, the A-T content in the non-coding (64.4%) is higher than in the coding (59.9%) regions. The A-T contents of the IR region (56.6%) in two cp genomes are identical but the A-T contents in the LSC (63.7% and 63.8%) and SSC regions (67.9% and 68.0%) are slightly different. Sixty-seven and seventy-three SSR loci which were repeated more than 10 times identified in the *P. stellatus* and *P. yatabeanus* cp genomes, respectively.

**Figure 1.**
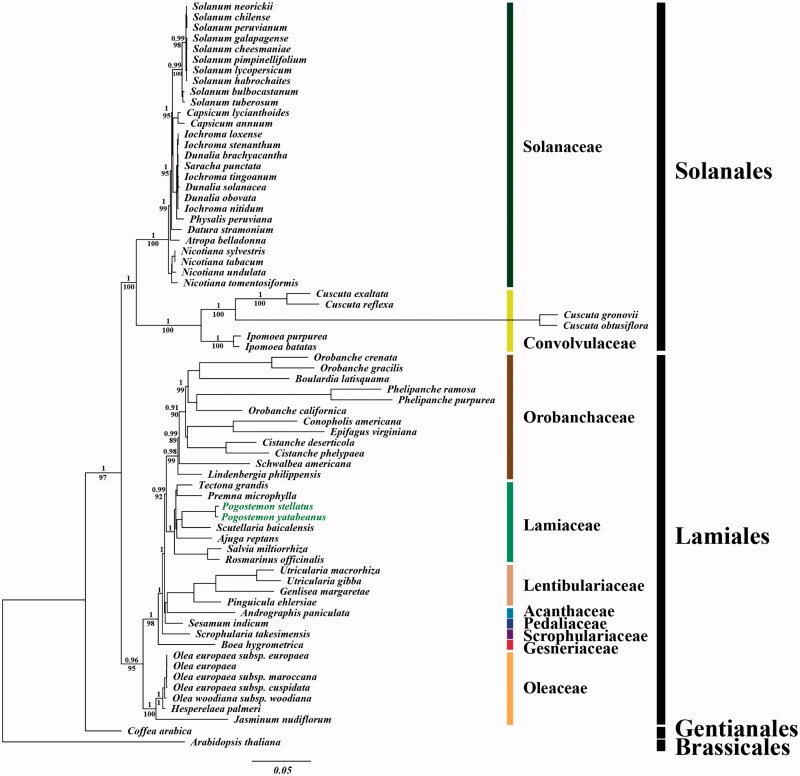
Chloroplast phylogenetic tree of the asterid I clade. A maximum-likelihood tree (−lnL= −578964.0368) inferred from analysis of alignment data containing 78 protein-coding genes in 70 chloroplast genome sequences by use of the GTR + Γ+I model. The numbers above and below each node indicate the Bayesian support percentages and bootstrap values, respectively. The scientific names with green color indicate two new sequences from *P. stellatus* (Lour.) Kuntze and *P. yatabeanus* (Makino) Press.

For the phylogenetic analysis, we assembled the 68 complete cp DNA sequences from the Solanales and Lamiales clade and two outgroup sequences from Gentianales and Brassicales clades. A total of 78 protein CDSs including *rrn* genes were aligned for the 70 analyzed taxa. The aligned data matrix consists of a total of 91,395bp. An ML tree was obtained with an − lnL = 578,964.0368 using the GTR + G + I base substitution model (Figure 1). Similar to previous studies, genus *Pogostemon* forms a monophyletic group with *Scutellaria baicalensis* in Scutellarioideae and this monophyletic group shows close relationship with *Ajuga reptans* in Ajugoideae (Ingrouille & Bhatti 1998; Steane et al. 2004; Scheen et al. 2010; Bendiksby et al. 2011).
